# Acute adverse events in cardiac MR imaging with gadolinium-based contrast agents: results from the European Society of Cardiovascular Radiology (ESCR) MRCT Registry in 72,839 patients

**DOI:** 10.1007/s00330-019-06171-2

**Published:** 2019-04-30

**Authors:** Johannes Uhlig, Christian Lücke, Rozemarijn Vliegenthart, Christian Loewe, Matthias Grothoff, Andreas Schuster, Philipp Lurz, Alexis Jacquier, Marco Francone, Antonia Zapf, Christoph Schülke, Daniel Thomas, Matthias Stefan May, Jens Bremerich, Joachim Lotz, Matthias Gutberlet, the ESCR MRCT Registry contributors, Daniel Thomas

**Affiliations:** 1 Department for Diagnostic and Interventional Radiology, University Medical Center Goettingen, Goettingen, Germany; 2 Department of Diagnostic and Interventional Radiology, University of Leipzig - Heart Centre, Strümpellstrasse 39, 04289 Leipzig, Germany; 3 Department of Radiology, Center for Medical Imaging, University Medical Center Groningen, Groningen, Netherlands; 4 Division of Cardiovascular and Interventional Radiology, Department of Biomedical Imaging and Image-Guided Therapy, Medical University of Vienna, Vienna, Austria; 5 Department of Cardiology and Pneumology, University Medical Center Goettingen, Goettingen, Germany; 6 Department of Cardiology, University of Leipzig - Heart Centre, Leipzig, Germany; 7 Department of Radiology, University of Marseille Méditerranée CHU la Timone, Marseille, France; 8 Department of Radiological, Oncological and Pathological Sciences, Sapienza University of Rome, Rome, Italy; 9 Institute for Medical Statistics, University Medical Center Goettingen, Goettingen, Germany; 10 Department of Clinical Radiology, University Muenster, Munster, Germany; 11 Department of Radiology, University of Bonn, Bonn, Germany; 12 Department of Radiology, University Hospital Erlangen, Erlangen, Germany; 13 Radiology Department, University Hospital Basel, Basel, Switzerland; 14 German Cardiovascular Research Center (DZHK), Partner site, Goettingen, Germany

**Keywords:** Adverse drug event, Gadolinium, Cardiac imaging techniques, MRI

## Abstract

**Objectives:**

To assess the incidence of acute adverse events (AAEs) in gadolinium-enhanced cardiac magnetic resonance (CMR) imaging.

**Methods:**

Gadolinium-based contrast agent (GBCA)–enhanced CMR data from the multinational, multicenter European Society of Cardiovascular Radiology MRCT Registry was included. AAE severity was classified according to the American College of Radiology Manual on Contrast Media (mild, moderate, severe). Multivariable generalized linear mixed effect models were used to assess the likelihood of AAEs in various GBCA, adjusting for pharmacological stressor, main indications (i.e., suspected or known coronary artery disease or myocarditis), age, sex, and submitting center as a random effect.

**Results:**

In the study population of 72,839 GBCA-enhanced CMRs, a total of 260 AAEs were reported (0.36%), with a minority of severe AAEs (*n* = 24, 0.033%). Allergic-like AAEs were less likely than physiologic AAEs (29% versus 71%). Patients without pharmacological stress imaging had a lower AAE rate (0.22%) compared to stress imaging (0.75%), with the highest AAE rates for regadenoson (2.95%). AAE rates also varied by GBCA subtype (overall *p* < 0.001). There was significant interaction between GBCA and pharmacological stressor (interaction *p* = 0.025), with AAE rates ranging between 0 and 10% for certain GBCA/stressor combinations. There was further marginal evidence that higher GBCA volume was associated with higher AAE incidence (OR = 1.02, *p* = 0.05).

**Conclusion:**

GBCA-enhanced CMR imaging demonstrates low AAE rates comparable to those of other body regions. AAE likelihood correlates with GBCA subtype, pharmacological stressor, and imaging indication. Intravenous fluid administration in patients with cardiac impairment might contribute to these findings.

**Key Points:**

*• Acute adverse event rates in cardiac magnetic resonance (CMR) imaging with gadolinium-based contrast agents (GBCAs) are low (0.36%), especially for severe adverse events (0.033%).*

*• Mild and moderate adverse events are more frequent during stress CMR imaging.*

*• Physiologic AAEs are more common than allergic AAEs in CMR imaging.*

**Electronic supplementary material:**

The online version of this article (10.1007/s00330-019-06171-2) contains supplementary material, which is available to authorized users.

## Introduction

Gadolinium-based contrast agents (GBCAs) are considered safe in magnetic resonance (MR) imaging with acute adverse event rates reported to be ranging from 0.04 to 2.2% [1–7].

Differences in adverse event rates by anatomical region might originate not only from distinct imaging algorithms but also from different propensities depending on the patient’s underlying pathology. In this context, cardiac imaging is of special interest, with a variety of indications ranging from cardiac viability assessment in older multimorbid patients to myocarditis imaging in primarily younger and healthier patients [8–10].

Moreover, cardiac MR (CMR) imaging for ischemic heart disease is routinely performed with pharmacological stressors, which might increase the incidence of acute adverse events in general, and GBCA-related acute adverse events in specific [11].

So far, acute adverse events in CMR imaging have been systematically evaluated in the European Cardiovascular Magnetic Resonance (EuroCMR) Registry [12]. Adverse event rates ranged from 0.17% in a sample of 17,767 patients to 0.12% in a sample of 37,788 patients [5, 13]. However, there is no comprehensive literature evaluating acute adverse events in GBCA-enhanced cardiac MR imaging with statistical adjustment for potential confounders, such as stress test imaging, main indications, age, and sex.

Therefore, the aim of the current study was to evaluate data originating from the multicenter, multinational cardiac MRCT Registry of the European Society of Cardiovascular Radiology (ESCR) to assess the likelihood of gadolinium-associated acute adverse events in cardiac MR imaging.

## Methods

This retrospective study was performed in accordance with the Declaration of Helsinki and received institutional review board approval (Leipzig University, No. 131/17-ek).

The data source of this study is the multinational, multicenter ESCR MRCT Registry, which includes imaging studies submitted between 2013 and 2016. Using a standardized online questionnaire, the physicians responsible for CMR imaging prospectively provided mandatory information on patient characteristics, indications, diagnoses, imaging techniques, contrast media application, and occurrence of acute adverse events (reported as the most severe event for each patient). Information on GBCA concentration and volume was non-mandatory. MRCT Registry users were unaware that data was utilized to assess acute adverse event incidences, thereby minimizing potential reporting biases.

Only CMR scans with intravenous administration of GBCA were included. GBCA molecular structure was classified as cyclic or linear, its ionic properties as ionic or non-ionic, and its thermodynamic chelate stability by log *K*_therm_ [14]. Covariates included pharmacological stressors, main indication, GBCA volume and concentration, gender, and age. Imaging indications rather than diagnoses were evaluated to avoid reverse causation, since acute adverse events might have influenced diagnoses (i.e., aborted examination or artifacts as a consequence of acute adverse events). Five administrations of gadoversetamide (Optimark^®^, Medtronic-Covidien) were excluded from analyses to avoid non-convergence of the statistical models due to a too small subgroup size.

### Outcomes

Acute adverse events (AAEs) were categorized as allergic-like or physiologic and classified as mild, moderate, or severe according to the American College of Radiology (ACR) Manual on Contrast Media [15]. As the AAE *dyspnea* is not specified according to the ACR, it was considered as physiologic given our study cohort with potential cardiac impairment. Hypersensitive AAE included urticaria and hives, as well as those categorized as *hypersensitive* without further detail by the treating physician. AAE category and severity are summarized in Table 2. Primary study outcome was any acute adverse event.

### Statistics

Descriptive statistics provided are absolute numbers and percent for categorical variables, and mean and standard deviation (SD) for continuous variables. For evaluation of the outcome *acute adverse event*, multivariable logistic regression models were fit with variable selection based on univariate significance and clinical knowledge. A generalized linear mixed effects model (GLMM) with submitting institution as a random effect was chosen to account for institutional differences in patient populations. A priori, a test for multiplicative interaction between pharmacological stressor and GBCA was planned. For sensitivity analyses, the outcomes *allergic-like AAE* and *physiologic AAE* were evaluated. For statistical modeling, the largest GBCA subgroup of patients receiving gadobutrol was chosen as reference. All statistics were performed using R (version 3.3.2) and R Studio (version 1.0.44) [16, 17].

All *p* values provided are two-sided. An alpha level of 0.05 was chosen for statistical significance.

## Results

### Baseline characteristics

**Fig. 1:**
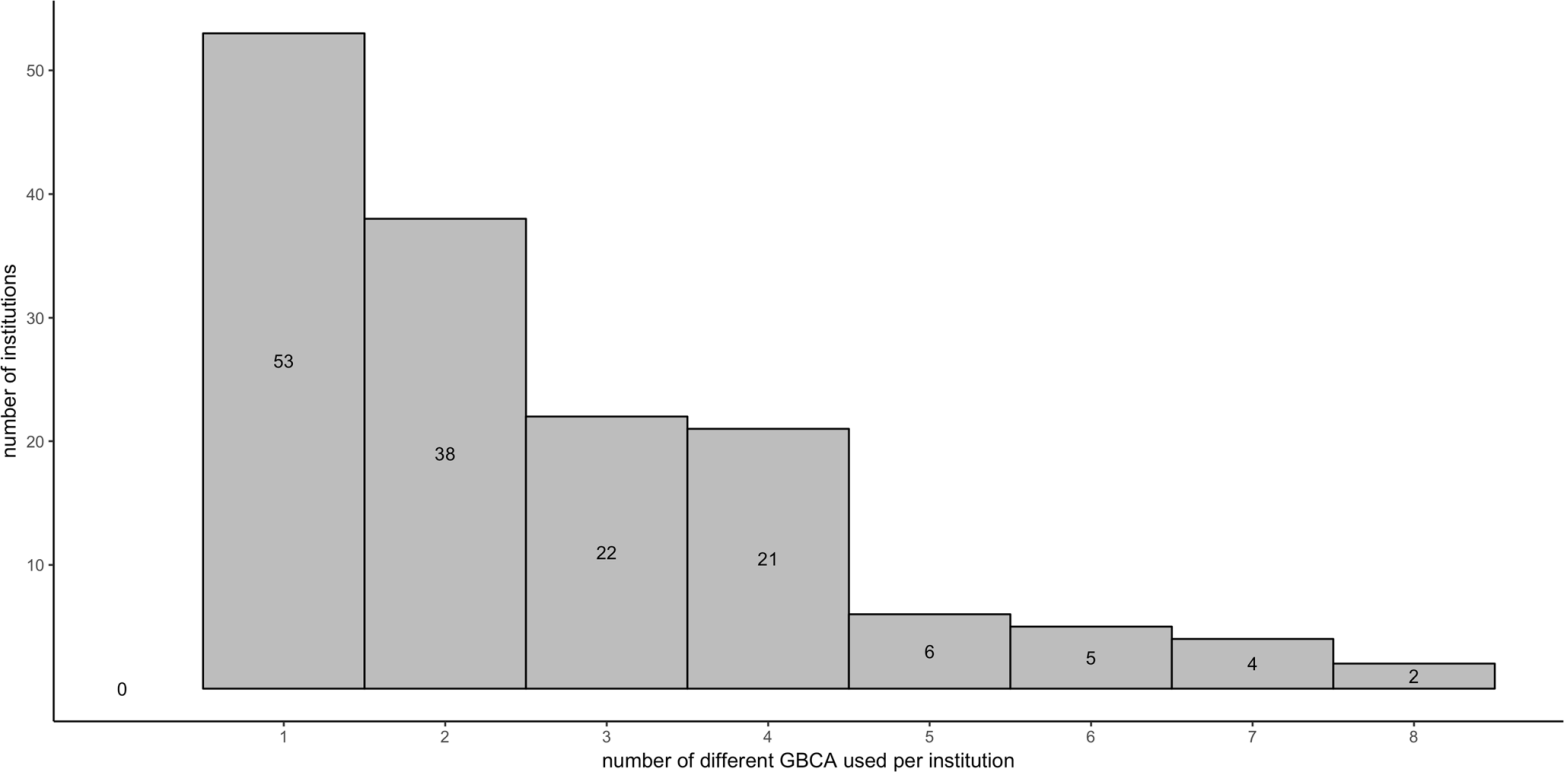
Frequency of different GBCAs used by each participating institution

A total of 72,839 CMR studies submitted to the ESCR MRCT Registry between 2013 and 2016 fulfilled the inclusion criteria. CMR studies were performed in 152 distinct participating centers, the majority of which used either one or two different GBCA subtypes (*n* = 53 [34.9%] and *n* = 38 [25%], respectively). Figure 1 plots the number of different GBCA subtypes used by each institution.

Most imaging studies were performed without pharmacological stressor (74.5%). Older patients and those with known or suspected coronary artery disease (CAD) as the main imaging indication were more likely to receive pharmacological stressors, as shown in Table 1. The most frequently used GBCA was gadobutrol (Gadovist^®^, Bayer Healthcare; *n* = 40,620, 56%), followed by gadoteric acid (Dotarem^®^, Guerbet; *n* = 14,257, 20%). Baseline parameters stratified by GBCA subtype are provided in the supplemental material.

**Table 1:** Baseline characteristics of included patients

	Total (*N* = 72,839)	No stress (*N* = 54,285, 74.5%)	Stress (*N* = 18,554, 25.5%)	Adenosine (*N* = 16,921, 91.2%)	Regadenoson (*N* = 1151, 6.2%)	Dobutamine (*N* = 482, 2.6%)
Age (years)	52 (± 19)	49 (± 20)	62 (± 14)	61 (± 14)	63 (± 12)	64 (± 13)
Gender, *n* (%)						
Male	47,040 (65)	34,506 (64)	12,534 (68)	11,397 (67)	812 (71)	325 (67)
Female	25,799 (35)	19,779 (36)	6020 (32)	5524 (33)	339 (29)	157 (33)
GBCA, *n* (%)						
Gadobutrol (i.e., Gadovist^®^)	40,620 (56)	30,366 (56)	10,254 (55)	9383 (55)	680 (59)	191 (40)
Gadoteric acid (i.e., Dotarem^®^)	14,257 (20)	10,780 (20)	3477 (19)	3014 (18)	430 (37)	33 (7)
Gadobenate (i.e., MultiHance^®^)	7092 (10)	4768 (9)	2324 (13)	2182 (13)	16 (1)	126 (26)
Gadopentetate (i.e., Magnevist^®^)	5624 (8)	4120 (8)	1504 (8)	1391 (8)	16 (1)	97 (20)
Gadoteridol (i.e., ProHance^®^)	2994 (4)	2290 (4)	704 (4)	669 (4)	6 (1)	29 (6)
Gadodiamide (i.e., Omniscan^®^)	2252 (3)	1961 (4)	291 (2)	282 (2)	3 (0)	6 (1)
GBCA volume (ml)						
Mean (SD)	18.90 (± 10.23)	18.36 (± 9.97)	20.08 (± 10.7)	19.34 (± 10.1)	30.2 (± 14.1)	21.06 (± 8.8)
Missing, *n* (%)	44,394 (61)	34,774 (64.1)	9620 (51.9)	8772 (51.8)	578 (50.2)	270 (56.0)
GBCA concentration (mmol/kg)						
Mean (SD)	0.17 (± 0.06)	0.17 (± 0.05)	0.16 (± 0.07)	0.16 (± 0.07)	0.13 (± 0.1)	0.18 (± 0.1)
Missing, *n* (%)	43,630 (60)	34,283 (63.2)	9347 (50.4)	8515 (50.3)	578 (50.2)	254 (52.7)
Main indication, *n* (%)						
Known CAD	12,936 (18)	6587 (12)	6349 (34)	5758 (34)	448 (39)	143 (30)
Suspected CAD	15,016 (21)	4427 (8)	10,589 (57)	9677 (57)	606 (53)	306 (63)
Suspected/known CMP	12,856 (18)	12,448 (23)	408 (2)	376 (2)	28 (2)	4 (1)
Suspected/known myocarditis	16,040 (22)	15,767 (29)	273 (1)	242 (1)	26 (2)	5 (1)
Others	15,991 (22)	15,056 (28)	935 (5)	868 (5)	43 (4)	24 (5)

*CAD* coronary artery disease, *CMP* cardiomyopathy, *GBCA* gadolinium-based contrast agent, *SD* standard deviation

Final diagnoses based on CMR imaging were cardiomyopathy in 10,938 patients (15%), CAD in 9939 patients (13.6%) and exclusion of CAD in 9409 patients (12.9%), myocardial infarction in 8228 patients (11.3%), valve disease in 8174 patients (11.2%), myocarditis in 6302 patients (8.7%), and miscellaneous diagnoses in 23,455 patients. In another 1591 patients (2.2%), CMR imaging revealed no pathological findings. CMR examinations were aborted in 30 patients due to AAEs yielding no final imaging diagnosis.

Full data on GBCA volume (ml) and molal concentration (mmol/kg) was available for 18,849 imaging studies (25.8%). The mean GBCA volume was 18.9 ml (± 10.2), with higher volumes reported in the regadenoson subgroup (30.2 ml). Thirty-seven percent of these patients received gadoteric acid. The mean volume administered showed a variation depending on the contrast agent used: the highest volumes for gadoteridol (33.8 ± 7.9 ml), gadopentetate (27.8 ± 12.9 ml), and gadoteric acid (27.5 ± 9.9 ml) compared to gadobutrol (13.4 ± 5 ml), gadobenate (17.3 ± 5.3 ml), or gadodiamide (18.6 ± 9 ml). The mean gadolinium concentration was 0.17 ± 0.06 mmol/kg bodyweight, with lower concentration for gadodiamide (0.15 ± 0.05 mmol/kg) and higher concentrations for gadoteridol (0.19 ± 0.04 mmol/kg). This suggests that the majority of CMR examinations were performed using a 1.5 or double dose of intravenous Gd-DTPA.

### Adverse events

In total, 260 acute adverse events (0.36%) were reported. According to the ACR criteria [15], the majority of AAEs were classified as mild (*n* = 104 [absolute, 0.143%; relative, 40%]) or moderate (*n* = 132 [absolute, 0.181%; relative, 50.8%]), and only few as severe (*n* = 24 [absolute, 0.033%; relative, 9.2%]). Allergic-like AAEs were less likely than physiologic AAEs (*n* = 76 [absolute, 0.104%; relative, 29%] versus *n* = 184 [absolute, 0.253%; relative, 71%]). The most frequent acute adverse event was dyspnea (*n* = 88, 33.8%), followed by hypersensitive reactions (*n* = 61, 23.5%) and emesis (*n* = 17, 6.5%).

The AAE rate was higher among patients receiving pharmacological stressors (*n* = 140/18,554, 0.75%) compared to non-stress imaging (*n* = 120/54,285, 0.22%, *p* < 0.001). Across different pharmacological stressor subgroups, patients receiving regadenoson had higher AAE rates (*n* = 34/1151, 2.95%), when compared to adenosine (*n* = 99/16,921, 0.59%) or dobutamine (*n* = 7/482, 1.45%), but the only seven severe AAEs (0.041%) associated with pharmacological stressors occurred in the adenosine group.

In addition to acute adverse events, ten extravasations of GBCA were reported: five in the non-stress group (0.009%), four in the adenosine group (0.024%), and one in the regadenoson group (0.087%). Table 2 shows frequencies of specific acute adverse events in the dataset.

**Table 2:** Incidence and type of acute adverse events

Category	Severity	Specific adverse event	Total (*N* = 72,839)	No stress (*N* = 54,285, 74.5%)	Stress (*N* = 18,554, 25.5%)	Adenosine (*N* = 16,921, 91.2%)	Regadenoson (*N* = 1151, 6.2%)	Dobutamine (*N* = 482, 2.6%)
Acute adverse events, *n* (%)								
No			72,579 (99.64)	54,165 (99.78)	18,414 (99.25)	16,822 (99.41)	1117 (97.05)	475 (98.55)
Yes			260 (0.36)	120 (0.22)	140 (0.75)	99 (0.59)	34 (2.95)	7 (1.45)
Adverse events, *n* (%)								
Physiologic adverse events (*n* = 184, 71%)	Mild	Back pain	2 (0.003)	1 (0.002)	1 (0.005)	–	1 (0.087)	–
	Mild	Emesis	17 (0.023)	11 (0.020)	6 (0.032)	5 (0.030)	–	1 (0.207)
	Mild	Heating	6 (0.008)	2 (0.004)	4 (0.022)	2 (0.012)	2 (0.174)	–
	Mild	Others	4 (0.005)	–	4 (0.022)	4 (0.024)	–	–
	Mild	Anxiety	17 (0.023)	4 (0.007)	13 (0.070)	8 (0.047)	2 (0.174)	3 (0.622)
	Moderate	Angina pectoris	13 (0.018)	4 (0.007)	9 (0.049)	7 (0.041)	2 (0.174)	–
	Moderate	Dyspnea	88 (0.121)	26 (0.048)	62 (0.334)	35 (0.207)	24 (2.085)	3 (0.622)
	Moderate	Symptomatic bradycardia	12 (0.016)	4 (0.007)	8 (0.043)	6 (0.035)	2 (0.174)	–
	Moderate	Symptomatic hypertension	2 (0.003)	2 (0.004)	–	–	–	–
	Moderate	Symptomatic hypotension	6 (0.008)	4 (0.007)	2 (0.011)	2 (0.012)	–	–
	Severe	Arrhythmia	13 (0.018)	11 (0.020)	2 (0.011)	2 (0.012)	–	–
	Severe	Renal failure	1 (0.001)	–	1 (0.005)	1 (0.006)	–	–
	Severe	Resuscitation	3 (0.004)	1 (0.002)	2 (0.011)	2 (0.012)	–	–
Allergic-like adverse events (*n* = 76, 29%)	Mild	Hypersensitive reaction	61 (0.084)	41 (0.076)	20 (0.108)	19 (0.112)	1 (0.087)	–
	Moderate	Respiratory adverse event	8 (0.011)	4 (0.007)	4 (0.022)	4 (0.024)	–	–
	Severe	Severe allergic reaction	7 (0.010)	5 (0.009)	2 (0.011)	2 (0.012)	–	–

### Statistical models for acute adverse events in GBCA-enhanced cardiac MR imaging

The final multivariable GLMM for AAEs of any severity included the following covariates: age, gender, pharmacological stressor, GBCA, and main indication. Age and gender were included in the model despite statistical non-significance at the chosen alpha level to adjust for potential confounding as described in the literature [3, 15]. Submitting institutions were considered as random effects in the GLMM to account for differences in the underlying patient population or AAE ascertainment methods. Results are presented in Table 3 and in the following paragraphs.

**Table 3:** Final multivariable logistic regression model for the outcome *acute adverse event* using a GLMM with submitting institution as random effect

Covariate	Molarity (mmol/ml)	Odds ratio	95% CI		*p* value
			Lower	Upper	
Age		0.992	0.985	1	0.064
Gender					
Male		Reference			
Female		1.089	0.839	1.415	0.52
MR stress test					
No stress test		Reference			
Adenosine stress test		2.311	1.559	3.426	< 0.001
Regadenoson stress test		3.922	2.258	6.81	< 0.001
Dobutamine stress test		4.134	1.7	10.052	0.002
GBCA					
Gadobutrol (i.e., Gadovist^®^)	1.0	Reference			
Gadobenate (i.e., MultiHance^®^)	0.5	1.546	0.88	2.715	0.13
Gadodiamide (i.e., Omniscan^®^)	0.5	0.734	0.192	2.811	0.651
Gadopentetate (i.e., Magnevist^®^)	0.5	1.711	0.947	3.092	0.075
Gadoteric acid (i.e., Dotarem^®^)	0.5	0.89	0.533	1.486	0.656
Gadoteridol (i.e., ProHance^®^)	0.5	3.58	1.832	6.995	< 0.001
Main indication					
Known CAD		Reference			
Suspected CAD		0.968	0.695	1.348	0.847
Suspected/known CMP		0.589	0.352	0.985	0.044
Suspected/known myocarditis		0.523	0.308	0.888	0.016
Other main indications		0.593	0.365	0.966	0.036

### Pharmacological stress imaging

Patients undergoing stress test imaging were more likely to develop acute adverse events of any severity compared to non-stress imaging: patients receiving adenosine, regadenoson, or dobutamine were more likely to exhibit acute adverse events (OR = 2.31, 3.92, and 4.13, respectively; *p* < 0.001, *p* < 0.001, and *p* = 0.002, respectively). As shown in Table 2, adverse event severity varied across pharmacological stressors: while severe adverse events only occurred in adenosine stress imaging (*n* = 7/16,921, 0.041%), the majority of adverse events in regadenoson stress imaging were of moderate severity (*n* = 29/1151 [absolute, 2.52%; relative, 85.3%]) and mainly attributable to dyspnea (*n* = 24).

### GBCA

In comparison to gadobutrol, patients receiving gadobenate, gadopentetate, and gadoteridol were more likely to develop acute adverse events, and those receiving gadodiamide and gadoteric acid were less likely to develop acute adverse events. Findings reached statistically significance at the chosen alpha level for gadoteridol (OR = 3.58, 95% CI = 1.83–6.99, *p* < 0.001), independent from the following covariates: pharmacological stressor, main indications, age, and gender.

There was a statistically significant multiplicative interaction between pharmacological stressors and GBCAs (*p* = 0.0252 for the 15-degree-of-freedom interaction test between GBCA and pharmacological stressor). For example, there was a ninefold increase in gadobutrol-associated adverse event rates comparing adenosine to regadenoson stress imaging (0.55% versus 4.56%), while gadoteric acid–associated adverse event rates were balanced (0.3% versus 0.47%).

Molecular structure, ionic properties, and thermodynamic chelate stability of GBCA did not influence acute adverse events at the chosen alpha level (cyclic versus linear molecular structure: univariate OR = 0.76, 95% CI = 0.51–1.15, *p* = 0.197; ionic versus non-ionic GBCA: univariate OR = 1.16, 95% CI = 0.8–1.69, *p* = 0.43; log *K*_therm_: univariate OR = 1.04, 95% CI = 0.93–1.15, *p* = 0.492, respectively, using GLMM with submitting institution as random effect). Acute adverse event rates across different GBCAs with corresponding molecular structure, ionic properties, and thermodynamic stability, as well as pharmacological stressors, are presented in Table 4. Specific acute adverse events for each GBCA subtype are further detailed in the supplemental material.

**Table 4:** Acute adverse events by stress imaging and pharmacological stressor

	Molarity (mmol/ml)	Molecular structure	Ionic/non-ionic	Thermodynamic chelate stability (log *K*_therm_)	Total (*N* = 72,839)	No stress (*N* = 54,285, 74.5%)	Stress (*N* = 18,554, 25.5%)	Adenosine (*N* = 16,921, 91.2%)	Regadenoson (*N* = 1151, 6.2%)	Dobutamine (*N* = 482, 2.6%)
All GBCA (% of patients)					260/72,839 (0.36)	120/54,285 (0.22)	140/18,554 (0.75)	99/16,921 (0.59)	34/1151 (2.95)	7/482 (1.45)
Gadobutrol (Gadovist^®^) (56%)	1.0	Cyclic	Non-ionic	21.8	143/40,620 (0.35%)	59/30,366 (0.19%)	84/10,254 (0.82%)	52/9383 (0.55%)	31/680 (4.56%)	1/191 (0.52%)
Gadoteridol (ProHance^®^) (4%)	0.5	Cyclic	Non-ionic	23.8	29/2994 (0.97%)	15/2290 (0.66%)	14/704 (1.99%)	11/669 (1.64%)	− /6 (0%)	3/29 (10.34%)
Gadoteric acid (Dotarem^®^) (20%)	0.5	Cyclic	Ionic	25.8	35/14,257 (0.25%)	24/10,780 (0.22%)	11/3477 (0.32%)	9/3014 (0.30%)	2/430 (0.47%)	− /33 (0%)
Gadobenate (MultiHance^®^) (10%)	0.5	Linear	Ionic	22.6	27/7092 (0.38%)	15/4768 (0.31%)	12/2324 (0.52%)	11/2182 (0.50%)	1/16 (6.25%)	− /126 (0%)
Gadopentetate (Magnevist^®^) (8%)	0.5	Linear	Ionic	22.1	23/5624 (0.41%)	6/4120 (0.15%)	17/1504 (1.13%)	14/1391 (1.01%)	− /16 (0%)	3/97 (3.09%)
Gadodiamide (Omniscan^®^) (3%)	0.5	Linear	Non-ionic	16.9	3/2252 (0.13%)	1/1961 (0.05%)	2/291 (0.69%)	2/282 (0.71%)	− /3 (0%)	− /6 (0%)

### Main indications

Compared to patients with known CAD, those with imaging indications (suspected/known cardiomyopathy, myocarditis, and other indications) were less likely to develop acute adverse events (OR = 0.59 [*p* = 0.044], OR = 0.52 [*p* = 0.016], and OR = 0.59 [*p* = 0.036], respectively).

### Sensitivity analyses

When analyzing the outcome allergic-like and physiologic AAEs separately, results for *any acute adverse event* were confirmed. For example, parameter estimates for gadoteridol were of comparable magnitude and direction (OR = 1.85 [*p* = 0.221] and OR = 4.98 [*p* < 0.001], respectively; see the supplemental material).

Further, GBCA volume and concentration were assessed in a subset of patients with available data (*n* = 18,849) as detailed in the supplemental material. In a multivariable model including GBCA volume, concentration, and GBCA subtype, there was marginal statistical evidence that higher GBCA volume was associated with a higher AAE likelihood (OR = 1.018 per 1 ml increment, *p* = 0.051). Although this effect did not persist after inclusion of the variable *stress test imaging*, it might be indicative that increased GBCA volume is associated with higher AAE likelihood. This association is further supported by a higher mean GBCA volume in the subgroup of severe physiologic AAEs (20.7 ml) compared to moderate AAEs (16.6 ml), as shown in the supplemental material.

## Discussion

The overall rate of acute adverse events (AAEs) in our multinational, multicenter cohort was 0.36%. This finding is in line with the literature on GBCA application in general and cardiac imaging in specific, ranging from 0.04 to 2.2% [1–7, 13, 18–21].

However, AAEs were more frequent compared to the EuroCMR Registry in 2011 and 2015, reporting AAE rates of 0.12–0.17%, while using the same ACR classification.

In particular, the number of moderate and severe AAEs was higher in the ESCR MRCT Registry: 40%, 50.8%, and 9.2% of the reported acute adverse events were mild, moderate, and severe, compared to 17.2% moderate and up to 6.3% severe adverse events in the EuroCMR Registry [6]. However, the latter study did not explicitly include cardiac stress test imaging. Differences in AAE rates could be further attributed to multiple factors, including diverging sample sizes and underlying population, selection bias related to discrepancies in documentation compliance and adverse event awareness, as well as differences in stress examinations and contrast agent usage. In this context, it has to be highlighted that the most frequent severe AAEs in our study were arrhythmias, which might be attributable to underlying cardiac disease in this specific patient population as well.

Our study population of 72,839 patients is the largest cohort evaluating AAEs in CMR imaging when compared to 37,788 [5] or 17,767 [13] patients in the EuroCMR Registry. Results on a lower AAE rate in the EuroCMR Registry might be related to a smaller cohort with less contribution—mainly academic centers and countries (57 versus 263 centers and 15 versus 32 countries). Further, our study evaluated a significantly larger proportion of male subjects (65% versus 62.2% male) [6].

In our study, the majority of patients demonstrated pathological CMR scans. While comparable data is unavailable from the EuroCMR Registry, one may speculate that the number of pathologic CMRs was lower than that in our cohort.

To the best of our knowledge, our study is the first to explicitly evaluate the interaction and occurrence of AAEs in combined stress CMR examinations with usage of GBCA. Only one EuroCMR Registry study provided information on stress CMR imaging, with severe AAEs (0.1%) reported exclusively in the group of patients receiving stress CMR imaging [22]. Still, EuroCMR Registry studies did not provide information on the exact pharmacological stressor used. In the ESCR MRCT Registry, regadenoson and dobutamine account for almost 10% of stress imaging studies, which are associated with more AAEs, i.e., due to a prolonged half-life and different modes of action as compared to adenosine [23].

There were considerable differences in the GBCA distribution of our study (Fig. 1) compared to the most recent EuroCMR Registry AAE study [6] with administration of gadopentetate (33.8%), gadobutrol (24.8%), gadodiamide (16.2%), and gadoteric acid (11.2%). However, the distribution of GBCA among vendors is substantially influenced by submitting centers and does not necessarily reflect the actual market shares in Europe.

Finally, discrepancies in documentation compliance and adverse event awareness might contribute to the higher number of AAEs observed in the radiological ESCR MRCT Registry as compared to the cardiology-initiated EuroCMR Registry: cardiologists might underrate or fail to report moderate AAEs such as dyspnea in the setting of stress CMR imaging.

## Adverse events risk factors

After multivariable statistical adjustment, pharmacological stressors and GBCA subtypes emerged as independent, significant predictors of acute adverse events. Patients subjected to stress imaging were more likely to develop acute adverse events than those without stress imaging. Across different pharmacological stressors, regadenoson and dobutamine showed an increased likelihood when compared to adenosine. In line with the recent literature, adverse event rates were higher among regadenoson and limited to mild and moderate events, mainly dyspnea [24, 25]. This difference could be attributed to the pharmacokinetic properties of regadenoson with a comparably long, tri-phasic half-life time [23]. However, increased respiratory adverse events could originate from selective regadenoson administration in patients with pulmonary diseases as well [26]. Since pulmonary diseases were not evaluated in the registry, residual confounding might have distorted our results.

Our findings on higher adverse event rates disagree with results from the EuroCMR Registry, showing lower adverse event rates in stress imaging [5, 13]. However, the authors did not statistically adjust for potential confounders such as age, gender, and imaging indications.

Although not the main aim of our study, gadoteridol had a higher likelihood than gadobutrol to be associated with acute adverse events. Disparities in GBCA molecular structure and chelate stability were suggested as potential explanations, supported by a recent meta-analysis showing increased AAE risk for linear ionic GBCA [3, 14, 27, 28]. The missing statistical influence of GBCA molecular properties in our study might be attributable to the overall low number of AAEs and consecutively low statistical power in certain subgroups. Still, comparisons between the GBCA subtypes were adequately powered to detect very small effect sizes at 90–95% power with an alpha level of 0.05, even considering the smallest GBCA strata [29]. Concerns that imbalances in GBCA strata sizes, ranging from approximately 2250 to 40,000 patients, biased our results are not supported by the current statistical literature [30].

Interestingly, GBCA-associated adverse events varied across pharmacological stressors. For example, gadobutrol-associated adverse event rates ranged from 0.52% for adenosine to 4.56% for regadenoson, while event rates were balanced for gadoteric acid (0.3% versus 0.47%). This might be explained by a potentiation of contrast media–associated acute events, depending on different pharmacological stressors, but has not been described in the literature so far.

Although not statistically significant, subgroup analyses suggest that higher GBCA volume might contribute to our findings. For example, higher GBCA volumes were evident in the regadenoson subgroup with high AAE incidence. Further, severe AAEs were mainly categorized as physiologic and received larger GBCA volumes compared to other strata. Especially considering our patient cohort with known or suspected cardiac impairment, intravenous fluid administration might intuitively correlate with AAE incidence, as described for patients with acute decompensated heart failure [31]. We aim to prospectively collect additional data on the total amount of intravenous fluid administration with updated versions of the MRCT Registry to further elucidate these pathomechanisms.

In contrast to earlier studies, we analyzed routinely used GBCAs and statistically adjusted for confounders. Differences in the underlying patient population or varying AAE ascertainment methods across the submitting centers were considered in statistical analyses. Reporting on AAEs in the context of CMR imaging was standardized in the ESCR MRCT Registry and completed by physicians. Finally, our results proved robust upon sensitivity analyses.

Still, our study is not devoid of limitations. Information on GBCA concentration and volume were non-mandatory and missing in the majority of cases, which might have biased results on GBCA-specific AAE profiles. There was no information on long-term follow-up, limiting the validity for late-onset adverse events such as nephrogenic systemic fibrosis [32]. Despite a large overall sample size, single stressor and GBCA subgroups might be undersampled to promote generalizable conclusions. The MRCT Registry was not specifically designed to evaluate GBCA safety, and selection biases must be considered regarding the non-random fashion in which stressor and contrast agents were administered. For example, cardiac imaging for cardiomyopathy was conducted mainly without pharmacological stressors, whereas stress imaging was performed in the majority of patients with suspected or known CAD. Furthermore, patients receiving regadenoson might have been selected due to underlying pulmonary diseases, thus explaining a higher rate of respiratory acute adverse events in the regadenoson subgroup. The proposed interactions between stressor and GBCA need therefore to be validated in randomized studies, controlling for underlying clinical conditions. Moreover, results on GBCA-specific AAE rates might be biased by selective data collection and reporting due to the registry design and the retrospective analysis of our study.

Finally, no information was obtained regarding the patients’ renal function and previous history of adverse reactions to contrast media, which may have biased our results.

## Conclusions

Our results suggest that gadolinium-based contrast agents are generally safe for application in cardiac MR imaging. Observed acute adverse event rates are within the range reported for general radiology and differ across GBCA, stress test imaging, and pharmacological stressor, as well as main indications. Higher volume intravenous fluid administration in patients with cardiac impairment might at least partially contribute to our findings. Further, pharmacological stressors might potentiate GBCA-associated adverse events. To the best of our knowledge, we provide the largest and most comprehensive study for acute adverse events in GBCA-enhanced cardiac MR imaging, evaluating a variety of routinely used GBCA in a multinational, multicenter setting.

## Electronic supplementary material

ESM 1 (DOCX 99 kb)

## Abbreviations

AAE: Acute adverse event
ACR: American College of Radiology
AUC: Area under the curve
CAD: Coronary artery disease
CMP: Cardiomyopathy
CMR: Cardiac magnetic resonance
EBCR: European Board of Cardiovascular Radiology
EuroCMR: European Cardiovascular Magnetic Resonance
GBCA: Gadolinium-based contrast agent
GLMM: Generalized linear mixed effects model
MR: Magnetic resonance
OR: Odds ratio
SD: Standard deviation
