# Supplementary material for: Acute adverse events in cardiac MR imaging with gadolinium-based contrast agents: results from the European Society of Cardiovascular Radiology (ESCR) MRCT Registry in 72,839 patients
Source: Eur Radiol. 2019 Apr 30;29(7):3686–95. doi: 10.1007/s00330-019-06171-2 (PMC6554260; doi:10.1007/s00330-019-06171-2)
Supplement: Supplementary file 1 — (DOCX 99 kb) [file 330_2019_6171_MOESM1_ESM.docx]

**Supplemental material**

**Supplemental Table 1:** GLMM for the outcome „allergic-like AAE“ with submitting institution as random effect.

| **Covariate** | **Molarity [mml/ml]** | **Odds Ratio** | **95% CI (lower-upper)** | | **p-value** |
| --- | --- | --- | --- | --- | --- |
| **age** |  | 0.981 | 0.968 | 0.994 | 0.005 |
| **gender** |  |  |  |  |  |
| male |  | reference |  |  |  |
| female |  | 1.261 | 0.79 | 2.013 | 0.331 |
| **MR stress test** | | | | | |
| No stress test |  | reference |  |  |  |
| adenosine stress test |  | 1.879 | 0.943 | 3.745 | 0.073 |
| regadenoson stress test |  | 1.216 | 0.152 | 9.703 | 0.854 |
| dobutamine stress test |  | 0 | 0 | Inf | 0.986 |
| **GBCA** | | | | | |
| Gadobutrol (i.e. Gadovist®) | 1.0 | reference |  |  |  |
| Gadobenate (i.e. Multihance®) | 0.5 | 2.023 | 1.033 | 3.962 | 0.04 |
| Gadodiamide (i.e. Omniscan®) | 0.5 | 0.47 | 0.062 | 3.537 | 0.463 |
| Gadopentetate (i.e. Magnevist®) | 0.5 | 1.033 | 0.39 | 2.732 | 0.948 |
| Gadoteric acid (i.e. Dotarem®) | 0.5 | 0.872 | 0.436 | 1.741 | 0.697 |
| Gadoteridol (i.e. Prohance®) | 0.5 | 1.849 | 0.691 | 4.945 | 0.221 |
| **Main indication** | | | | | |
| known CAD |  | reference |  |  |  |
| suspected CAD |  | 0.743 | 0.364 | 1.52 | 0.416 |
| suspected/known CMP |  | 0.749 | 0.312 | 1.797 | 0.517 |
| suspected/known Myocarditis |  | 0.8 | 0.341 | 1.875 | 0.607 |
| other main indications |  | 0.642 | 0.265 | 1.556 | 0.327 |

**Supplemental Table 2:** Multivariable GLMM for the outcome “physiologic AAE” with submitting institution as random effect.

| **Covariate** | **Molarity [mml/ml]** | **Odds Ratio** | **95% CI (lower-upper)** | | **p-value** |
| --- | --- | --- | --- | --- | --- |
| **age** |  | 0.999 | 0.989 | 1.009 | 0.892 |
| **gender** |  |  |  |  |  |
| male |  | reference |  |  |  |
| female |  | 1.025 | 0.749 | 1.405 | 0.876 |
| **MR stress test** | | | | | |
| No stress test |  | reference |  |  |  |
| adenosine stress test |  | 2.613 | 1.632 | 4.185 | <0.001 |
| regadenoson stress test |  | 4.023 | 2.193 | 7.377 | <0.001 |
| dobutamine stress test |  | 5.497 | 2.154 | 14.029 | <0.001 |
| **GBCA** | | | | | |
| Gadobutrol (i.e. Gadovist®) | 1.0 | reference |  |  |  |
| Gadobenate (i.e. Multihance®) | 0.5 | 1.393 | 0.652 | 2.976 | 0.392 |
| Gadodiamide (i.e. Omniscan®) | 0.5 | 0.857 | 0.154 | 4.768 | 0.86 |
| Gadopentetate (i.e. Magnevist®) | 0.5 | 2.004 | 0.976 | 4.117 | 0.058 |
| Gadoteric acid (i.e. Dotarem®) | 0.5 | 0.931 | 0.488 | 1.776 | 0.829 |
| Gadoteridol (i.e. Prohance®) | 0.5 | 4.978 | 2.139 | 11.588 | <0.001 |
| **Main indication** | | | | | |
| known CAD |  | reference |  |  |  |
| suspected CAD |  | 1.029 | 0.708 | 1.496 | 0.881 |
| suspected/known CMP |  | 0.519 | 0.269 | 1 | 0.05 |
| suspected/known Myocarditis |  | 0.328 | 0.154 | 0.699 | 0.004 |
| other main indications |  | 0.554 | 0.307 | 1.001 | 0.05 |

| **Supplemental Table 3:** Baseline characteristics of included patients stratified by GBCA subtype. | | | | | | | | | | |
| --- | --- | --- | --- | --- | --- | --- | --- | --- | --- | --- |
|  | **Total**  **No. 72,839** | **Gadobutrol (i.e. Gadovist®)**  **No. 40,620** | **Gadobenate (i.e. Multihance®)**  **No. 7,092** | **Gadodiamide (i.e. Omniscan®)**  **No. 2,252** | | **Gadopentetate (i.e. Magnevist®)**  **No. 5,624** | | **Gadoteric acid (i.e. Dotarem®)**  **No. 14,257** | **Gadoteridol (i.e. Prohance®)**  **No. 2,994** | |
| **age** | 52 (± 19) | 51 (± 20) | 56 (± 18) | 47 (± 23) | | 54 (± 17) | | 53 (± 19) | 55 (± 17) | |
| **gender** | | | | | | | | | | |
| male | 47,040 (65%) | 26,025 (64%) | 4,749 (67%) | 1,442 (64%) | | 3,605 (64%) | | 9,324 (65%) | 1,895 (63%) |  |
| female | 25,799 (35%) | 14,595 (36%) | 2,343 (33%) | 810 (36%) | | 2,019 (36%) | | 4,933 (35%) | 1,099 (37%) |  |
| **main indication** | | | | | | | | | | |
| known CAD | 12,936 (18%) | 6,392 (16%) | 1,906 (27%) | 273 (12%) | 1,013 (18%) | | 2,793 (20%) | | 559 (19%) | |
| suspected CAD | 15,016 (21%) | 8,231 (20%) | 1,603 (23%) | 355 (16%) | 1,453 (26%) | | 2,740 (19%) | | 634 (21%) | |
| suspected/known CMP | 12,856 (18%) | 7,085 (17%) | 1,392 (20%) | 523 (23%) | 780 (14%) | | 2,594 (18%) | | 482 (16%) | |
| suspected/known Myocarditis | 16,040 (22%) | 8,037 (20%) | 1,275 (18%) | 266 (12%) | 1,667 (30%) | | 4,013 (28%) | | 782 (26%) | |
| others | 15,991 (22%) | 10,875 (27%) | 916 (13%) | 835 (37%) | 711 (13%) | | 2,117 (15%) | | 537 (18%) | |
| **GBCA volume** [ml] | | | | | | | | | | |
| Mean (SD) | 18.90 (±10.23) | 13.39 (±5.00) | 17.29 (±5.34) | 18.64 (±9.03) | 27.82 (±12.87) | | 27.49 (±9.87) | | 33.83 (±7.93) | |
| Missing | 44,394 (60.95%) | 25,352 (62.41%) | 4,291 (60.50%) | 1,393 (61.86%) | 2,396 (42.60%) | | 8,912 (62.51%) | | 2,050 (68.47%) | |
| **GBCA concentration** [mmol/kg] | | | | | | | | | | |
| Mean (SD) | 0.17 (±0.06) | 0.16 (±0.05) | 0.17 (±0.08) | 0.15 (±0.05) | 0.18 (±0.07) | | 0.17 (±0.07) | | 0.19 (±0.04) | |
| Missing | 43,630 (59.90%) | 24,311 (59.85%) | 4,588 (64.69%) | 1,375 (61.06%) | 3,130 (55.65%) | | 8,159 (57.23%) | | 2,067 (69.04%) | |

**Supplemental Table 4:** adverse events by GBCA subtype (grey shade=physiologic adverse events / blue shade=allergic adverse events).

|  |  |  | **Total**  **No. 72,839** | **Gadobutrol (i.e. Gadovist®)**  **No. 40,620** | **Gadobenate (i.e. Multihance®)**  **No. 7,092** | **Gadodiamide (i.e. Omniscan®)**  **No. 2,252** | **Gadopentetate (i.e. Magnevist®)**  **No. 5,624** | **Gadoteric acid (i.e. Dotarem®)**  **No. 14,257** | **Gadoteridol (Prohance)**  **No. 2,994** |
| --- | --- | --- | --- | --- | --- | --- | --- | --- | --- |
| **category** | **severity** | **specific adverse event** |  |  |  |  |  |  |  |
| physiologic adverse ephsphysiolveph (n=184) | mild | back pain | 2 | 2 | 0 | 0 | 0 | 0 | 0 |
|  | mild | emesis | 17 | 7 | 2 | 0 | 0 | 3 | 5 |
|  | mild | heating | 6 | 4 | 0 | 0 | 0 | 1 | 1 |
|  | mild | Others | 4 | 4 | 0 | 0 | 0 | 0 | 0 |
|  | mild | anxiety | 17 | 7 | 0 | 0 | 4 | 1 | 5 |
|  | moderate | angina pectoris | 13 | 10 | 0 | 0 | 0 | 3 | 0 |
|  | moderate | dyspnea | 88 | 57 | 6 | 1 | 9 | 9 | 6 |
|  | moderate | symptomatic bradycardia | 12 | 2 | 4 | 0 | 1 | 2 | 3 |
|  | moderate | symptomatic hypertension | 2 | 0 | 0 | 0 | 1 | 1 | 0 |
|  | moderate | symptomatic hypotension | 6 | 2 | 0 | 0 | 0 | 2 | 2 |
|  | severe | arrhythmia | 13 | 9 | 0 | 0 | 1 | 1 | 2 |
|  | severe | renal failure | 1 | 0 | 1 | 0 | 0 | 0 | 0 |
|  | severe | resuscitation | 3 | 0 | 0 | 1 | 2 | 0 | 0 |
|  |  |  |  |  |  |  |  |  |  |
| allergic-like adverse events (n=76) | mild | hypersensitive reaction | 61 | 33 | 12 | 0 | 2 | 10 | 4 |
|  | moderate | respiratory adverse event | 8 | 2 | 2 | 0 | 2 | 2 | 0 |
|  | severe | severe allergic reaction | 7 | 4 | 0 | 1 | 1 | 0 | 1 |

**Supplemental Table 5.** Baseline characteristics of patients with data on GBCA volume and concentration.

|  | **No. 18,849** | |
| --- | --- | --- |
| **age** | 54 (± 19) | |
| **gender** | |  |
| male | 12,624 (67%) | |
| female | 6,225 (33%) | |
| **MR stress test** | |  |
| No stress test | 12,516 (66%) | |
| adenosine stress test | 5,632 (30%) | |
| regadenoson stress test | 512 (3%) | |
| dobutamine stress test | 189 (1%) | |
| **GBCA** | |  |
| Gadobutrol (i.e. Gadovist®) | 8,871 (47%) | |
| Gadobenate (i.e. Multihance®) | 2,035 (11%) | |
| Gadodiamide (i.e. Omniscan®) | 726 (4%) | |
| Gadopentetate (i.e. Magnevist®) | 1,925 (10%) | |
| Gadoteric acid (i.e. Dotarem®) | 4,704 (25%) | |
| Gadoteridol (i.e. Prohance®) | 588 (3%) | |
| **GBCA volume** [ml, mean±SD] | 19.66 (±10.52) | |
| **GBCA concentration** [mmol/ml, mean±SD] | 0.17 (±0.06) | |
| **Main indication** | |  |
| known CAD | 4,092 (22%) | |
| suspected CAD | 4,491 (24%) | |
| suspected/known CMP | 2,964 (16%) | |
| suspected/known Myocarditis | 4,033 (21%) | |
| other main indications | 3,269 (17%) | |

Supplemental Table 6: Logistic regression model for the outcome “acute adverse event” in the subset of patients with data on GBCA volume and concentration

|  | **OR** | **LCI** | **UCI** | **p value** |
| --- | --- | --- | --- | --- |
| **GBCA volume (ml)** | 1.018 | 1 | 1.037 | 0.051 |
| **GBCA concentration (mmol/kg)** | 0.003 | 0 | 0.259 | 0.011 |
| **GBCA** |  |  |  |  |
| Gadubutrol (i.e. Gadovist®) | reference |  |  |  |
| Gadoteric acid (i.e. Dotarem®) | 0.295 | 0.137 | 0.639 | 0.002 |
| Gadoteridol (i.e. Prohance®) | 1.272 | 0.429 | 3.77 | 0.665 |
| Gadobenate (i.e. Multihance®) | 0.824 | 0.401 | 1.695 | 0.6 |
| Gadodiamide (i.e. Omniscan®) | 0 | 0 | Inf | 0.971 |
| Gadopentetate (i.e. Magnevist®) | 1.232 | 0.644 | 2.359 | 0.528 |


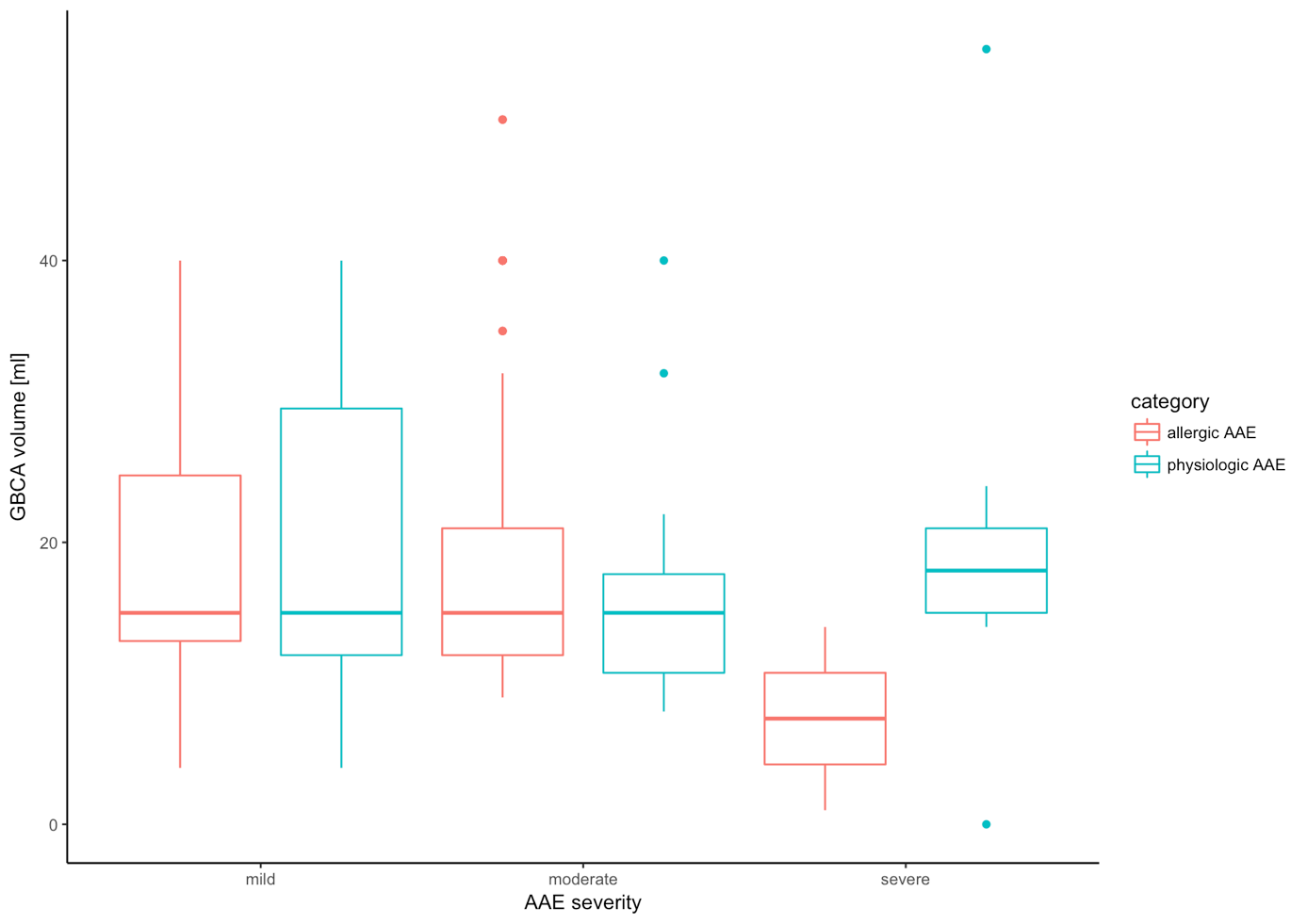


**Supplemental Figure 1:** GBCA volume in milliliter across different acute adverse event (AAE) severity for allergic and physiologic AAEs.
